# Imaging of Adverse Events Related to Checkpoint Inhibitor Therapy

**DOI:** 10.3390/diagnostics10040216

**Published:** 2020-04-13

**Authors:** Vanina Vani, Daniele Regge, Giovanni Cappello, Michela Gabelloni, Emanuele Neri

**Affiliations:** 1 Department of Translational Research, University of Pisa, Via Roma 67, 56126 Pisa, Italy; vanina.vani87@gmail.com (V.V.); mgabelloni@sirm.org (M.G.); emanuele.neri@med.unipi.it (E.N.); 2 Radiology Unit, Candiolo Cancer Institute, FPO-IRCCS, University of Torino, Candiolo, ‎10060 Turin, Italy; 3 Candiolo Cancer Institute, FPO-IRCCS, Candiolo, 10060 Turin, Italy; giovanni.cappello@ircc.it

**Keywords:** immune checkpoint inhibitors, therapy, toxicity, oncologic imaging

## Abstract

Immunotherapy with checkpoint inhibitors (ICIs) is becoming standard of practice for an increasing number of cancer types. ICIs enhance T-cell action against the cancer cells. By unbalancing the immune system ICIs may cause dysimmune toxicities, a series of disorders broadly defined immune-related adverse events (irAEs). IrAEs may affect any organ or apparatus and most frequently involve skin, colon, endocrine organs, liver, and lungs. Early identification and appropriate treatment of irAEs can improve patient outcome. The paper aims at reviewing mechanisms of the occurrence of irAEs, the importance of a proper diagnosis and the main pillars of therapy. To provide effective guidance to the comprehension of major irAEs imaging findings will be reviewed.

## 1. Introduction

In 2011 the U.S. Food and Drug Administration approved the first immune checkpoint inhibitor for cancer care and in the treatment of advanced melanoma. Subsequently, there was a rapid expansion to the use of immune checkpoint inhibitors (ICIs) and now these agents are standard therapies for several advanced stage malignancies including melanoma, non-small-cell lung cancer, and advanced renal and urothelial cancer (Table 1). Indeed, approximately one-third of patients with metastatic cancer now undergo therapy with ICIs at some point during their treatment. Unfortunately, immune-related adverse events (irAEs), such as colitis, dermatitis, pneumonia and endocrine complications are common during ICI therapy [1]. Imaging plays a significant role in the diagnosis and classification of irAEs and imaging findings are fundamental when deciding whether ICI therapy should be suspended and steroids and/or other support therapies administered [2,3,4,5]. However, since immunotherapy with ICIs has only recently been introduced in clinical practice, radiologists may not be familiar with radiological signs of irAEs and be unaware of the clinical implications of their findings, leading to the wrong choice of treatment. The aim of this article is therefore to review most important imaging finding of irAEs and their clinical significance in patients undergoing treatment with ICIs. 

A comprehensive search of electronic databases (PubMed and Google scholar) was conducted using the combination of the following key words: checkpoint inhibitor; immunotherapy; toxicity; complications; adverse events; imaging; radiology; PET/CT; CT; comparison; irAEs. In addition, textbooks concerning the subject were consulted. The bibliographies of relevant articles were additionally hand-searched for studies missed by the search strategy. Evidence from these data was critically analyzed and summarized to produce this paper with a broad overview about the most relevant irAEs in terms of frequency, severity and role of imaging.

## 2. Mechanisms Underlying irAEs

**Figure 1:**
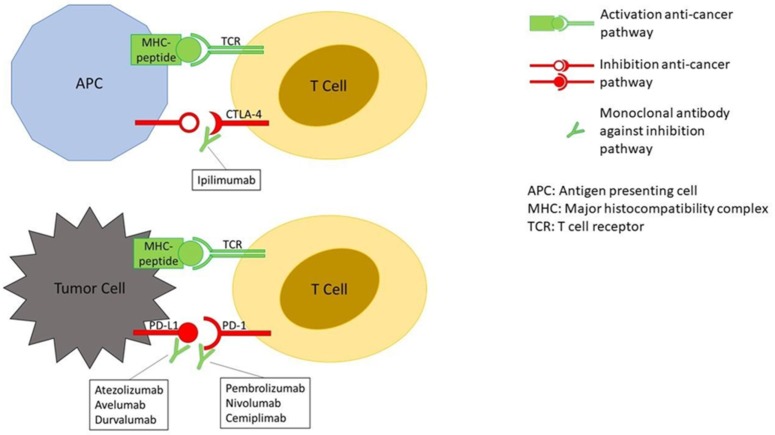
Mechanism of action of immune checkpoint inhibitors (ICIs). The binding together of MHC-peptide (peptide = tumor antigen) and TCR activates the anti-cancer pathway of T-cell–mediated immune response. The binding of CTLA-4 to its ligand and PD-1 to PD-L1, on the contrary, inhibit the anti-cancer pathway. The binding of monoclonal antibodies, such as ICIs, to CTLA-4, PD-1 or PD-L1 prevent the deactivation of the anti-cancer pathway.

The tumor microenvironment can overexpress molecules capable of inhibiting the T-cell–mediated immune response. Three molecules are central to this process, cytotoxic T-lymphocyte–associated antigen 4 (CTLA-4), programmed death 1 (PD-1), and its ligand PD-L1. The basic principle of immune checkpoint inhibitors (ICIs) is to block the CTLA-4 and the PD-1/PD-L1 pathways in order to enhance T-cell action against the cancer cells (Figure 1). 

Boosting the immune system leads to the so-called immune-related adverse events (irAEs) that may affect any system in the body, more frequently involving the skin, colon, lungs, endocrine glands, and liver [7]. As of March 2019, seven ICIs have been approved by the United States Food and Drug Administration (U.S. FDA) for the treatment of several tumors at different stages: one anti–CTLA-4 antibody (ipilimumab), three anti–PD-1 antibodies (pembrolizumab, nivolumab, and cemiplimab) and three anti–PD-L1 antibodies (atezolizumab, avelumab, and durvalumab) (Table 1). Most of the indications have been subsequently approved by the European Medicines Agency (EMA).

## 3. Grading Severity of irAEs

The grading scale for irAEs is mainly based on the Common Terminology Criteria for Adverse Events (CTCAE). However, when the delineations between two grades depends on patients’ recall, such as the number of stools in a day, the distinction between grade 2 and grade 3 could be challenging [8]. Approximately 90% of patients treated with anti-CTLA-4 and about 70% of those treated with any anti-PD-1 or anti–PD-L1 antibody have a toxicity, from mild to severe [7,9]. Toxicity incidence appears greater with anti-CTLA-4 because they interact with naive and memory T cells in the lymphatic tissues while anti-PD-1/PD-L1 interact with T-cell activity in peripheral tissues [10]. Furthermore, toxicities associated to Ipilimumab seem to be dose dependent, while those related to anti-PD-1/PD-L1 appear dose independent. Severe irAEs (grade 3 and 4), that may require hospitalization, occur in 10–42% of patients treated with anti-CTLA-4 ipilimumab, in 11–20% of patients treated with anti-PD-1 and in 1–9% of those treated with anti-PD-L1 agents. Grade 3–4 seems significantly higher for combined ICIs treatments [1]. Management of irAEs is generally based on the grade of severity and the clinical judgement. However, some recommendations propose histological examination in case of high-grade toxicity with significant diagnostic doubt [11]. 

## 4. Chronology of irAEs

Although the median time to onset of irAEs is 3–6 months from therapy initiation, the toxicity can occur earlier or even after one year from discontinuation of treatment [3]. According to Alessandrino et al., in 33.3% of cases irAEs occurred after therapy was discontinued, with a median time of 3 months between nivolumab discontinuation and imaging evidence of irAEs. The delayed effect of such complications underlines the importance of appropriate follow-up after discontinuation of therapy [12]. 

## 5. Treatment of Adverse Events

The American Society of Clinical Oncology (ASCO) Clinical Practice Guideline for diagnosis and management of specific organ system-based irAEs suggest not to discontinue ICI therapy in patients with grade 1 toxicities (except for some neurological, hematological, and cardiac toxicities). Most of grade 2 toxicities require the suspension of ICIs till the symptoms revert to grade 1 or less. Discontinuation of ICIs and the initiation of high-dose corticosteroid therapy is recommended in patients with grade 3 complications. Immunosuppressive therapy (i.e., infliximab) may be necessary for some refractory cases. In general, grade 4 toxicity should be managed with permanent withdrawal of ICIs (excluding endocrinopathies that are controlled by hormone replacement) [13]. 

### 5.1. Treatment of Gastrointestinal Toxicity

Diarrhea is the ICI’s adverse event that most frequently requires hospitalization [14]. The prevalence of severe colitis and severe diarrhea are reported respectively in 0.9% and 1.2% for anti PD-1/PD-L1; in 6.8% and 7.9% for ipilimumab and in 9.4% and 9.2% for combined treatment [15]. Life-threatening events like bloody diarrhea and perforation are rare [5,16]. There is no evidence of a correlation between the incidence of diarrhea/colitis and the type of tumor [15]. Diagnostic evaluation and suspension of ICIs is not recommended in grade 1 toxicity [3,13]. If grade 2–3 anti-PD-1/PD-L1-correlated colitis/diarrhea occur, a drug-free period is recommended while permanent withdrawal is recommended for grade 4 toxicity [13]. If colitis/diarrhea occur after anti-CTLA-4 or combined therapy (anti-CTLA-4 and anti-PD-1) a drug-free period for grade 2 and the termination of treatment for grade 3–4 are recommended [17]. The drug-free period is recommended until the symptoms are resolved and the eventual steroid treatment is completed. According to the results of retrospective studies patients who restarted ICIs had a 30–60% of risk of recurrence of the same irAE or of a new one; no clear benefit in terms of progression-free survival, or overall survival, were demonstrated [18,19,20,21]. For grade ≥ 2 diarrhea/colitis, American Society of Clinical Oncology (ASCO) and European Society for Medical Oncology (ESMO) guidelines recommend high dose steroid-therapy or infliximab in case of steroid-refractory diarrhea [3,13]. The timing of drug administration is also important. Abu-Sbeih et al. showed a better outcome in patients treated with early immunosuppressive therapy (infliximab, vedolizumab or both) compared to those who received a delayed immunosuppressive therapy [21]. For grade ≥ 2, computed tomography (CT) and colonoscopy with biopsy should be considered in order to exclude ulceration of the colonic mucosa, since in these patients steroid-refractory seems to be more frequent and infliximab should be started earlier [22]. More data are needed to clarify if a reintroduction of immunotherapy improves patient outcome in case of ICIs correlated diarrhea/colitis. Therefore, for now, the re-administration of ICIs after a drug-free period should be personalized to the patient considering his health status and other options of treatment.

### 5.2. Treatment of Hepatic Toxicity 

Hepatic toxicity grades are evaluated based on serum of aspartate aminotransferase, alanine transaminase, and bilirubin values [23]. Low grade toxicity generally manifests with asymptomatic laboratory abnormalities. Symptoms as anorexia, nausea, jaundice, and cutaneous or mucosal bleeding are typical of mild/severe grade of toxicity. In case of abnormal laboratory tests other causes of liver dysfunction as HBV, HAV, and HCV must be ruled out. Imaging should be considered to exclude metastatic disease, drug induced or viral hepatitis, and portal vein thrombosis [24,25]. According to the ASCO Clinical Practice Guideline and the National Comprehensive Cancer Network (NCCN) Guidelines Version 1.2020, for grade 1 toxicity, strict clinical and laboratory monitoring is suggested, without interrupting ICIs therapy [13,26]. For grade 2 toxicity, recommendations are to temporary suspend ICIs followed by administration of steroids or immunosuppressive therapy. For grade ≥ 3 toxicity, permanent discontinuation of ICIs and intravenous steroids are recommended. In steroid-refractory cases other immunosuppressive drugs have been suggested. A good response to treatment has been observed for mycophenolate mofetil [27,28] and for azathioprine [29]; no data are available for infliximab efficacy in patients with hepatic toxicity. Recurrence of irAEs has been observed during the gradual reduction or after the suspension of steroid therapy.

### 5.3. Treatment of Pulmonary Toxicity 

According to NCI-CTCAE 5.0 and ASCO guidelines ICIs-related pneumonitis has 4 grades of toxicity in relation to observed symptoms, daily life activity and the need for respiratory support [13,23]. The latter considers also diagnostic imaging including repeat CT of the thorax after 3–4 weeks from the onset of ICIs-related pneumonitis. Lung toxicity is rare but potentially fatal, so early diagnosis is fundamental, followed by suspension of immunotherapy and prompt treatment in severe cases. Patients with mild pneumonitis are closely monitored while those with severe pneumonitis receive supportive therapies (i.e., oxygen therapy) and high dose steroids [3,13]. The ASCO guidelines suggest withholding immunotherapy for any grade of pulmonary toxicity. Patient may resume the ICIs if there is 1-grade toxicity with radiographic evidence of improvement/resolution and if grade-2 toxicity drops to grade 1 or less. Grade 3 and 4 patients should interrupt therapy and be hospitalized [13]. A 4–6 weeks regimen of corticosteroids is suggested in moderate or severe pneumonitis; other immunosuppressive agents (i.e., infliximab, mycophenolate mofetil, cyclophosphamide) should be considered if patient is refractory to corticosteroid treatment [13]. 

In addition to typical findings of pneumonitis, sarcoid-like granulomatous reactions have been associated with ICIs. Such reactions can be asymptomatic or can manifest with cough, wheezing, fatigue, and chest pain. The diagnosis is challenging as they can mimic progression disease and there are no approved guidelines for their treatment. The therapy should be personalized to the patient in a multidisciplinary approach [30,31,32,33]. 

### 5.4. Treatment of Thyroid Toxicity 

Recent studies demonstrate that thyroiditis is the most frequent endocrine irAE. Furthermore, the incidence appears higher in patients receiving combined therapy (CTLA-4 + PD-1/PD-L1) than those treated with monotherapy (more common with anti-PD-1/PD-L1 than with anti-CTLA-4). Primary hypothyroidism (elevated TSH, normal or low FT4) shows greater incidence than hyperthyroidism (suppressed TSH and high normal or elevated FT4 and/or triiodothyronine). The latter seems to be a transient inflammation status of the thyroid gland that often switches in hypothyroidism [34,35,36]. According to NCI-CTCAE 5.0, hypothyroidism and hyperthyroidism ICIs-related have 4 grades of toxicity based on symptoms (abnormal heartbeat, increased sweating or feeling cold, tiredness or weakness, muscle aches, weight changes, mood changes and constipation) and activity of daily life; in addition, the ASCO guidelines considers the level of TSH to distinguish hypothyroidism grade 1 (TSH < 10 mIU/L) from grade 2 (TSH > 10 mIU/L). In general, the ASCO guidelines recommends to continue ICIs with close follow-up of thyroid function in case of grade 1 toxicity; for grade 2 toxicity patients may hold immunotherapy until symptoms resolve to baseline with the addition of thyroid hormone supplementation (if symptomatic hypothyroidism or persistent TSH > 10 mIU/L) or in addition of b-Blocker (if symptomatic hyperthyroidism). Nevertheless, various observational studies have showed that patients with grade 2 toxicity may be properly managed with supportive therapy (levothyroxine, b-blocker, and/or thionamide) without interrupting immunotherapy [37,38]. Grade 3–4 thyroiditis are rare [35,36]. The ASCO guidelines suggest withholding ICIs therapy until symptoms resolve. Appropriate supplementation is indicated for patients with hypothyroidism. In case of hyperthyroidism b-Blockers are indicated for symptom relief; moreover, for severe symptoms or concern for thyroid storm, hospitalization and the use of potassium iodide or thionamide should be considered. Finally, high dose corticosteroids are indicated in Graves’ ophthalmopathy [39].

## 6. Most Relevant Imaging Findings in irAEs

IrAEs often result in abnormalities that may be identified on CT, Magnetic Resonance Imaging (MRI), Ultrasound (US), or Fluorodeoxyglucose-Positron Emission Tomography (FDG-PET) performed for restaging and/or surveillance. Mekki et al. found that medical imaging (CT, MRI, US, plain radiographs, and FDG-PET/CT) were able to detect 74% of irAEs grade ≥ 2 in patients treated with anti-PD-1 [40]. Patients with ICIs-related toxicity may have a dynamic clinical course, and imaging is only a snapshot taken in a specific time point of an evolving clinical scenario. Moreover, immune-related adverse events may produce imaging findings before the appearance of clinical symptoms, allowing early change of therapy [41]. Tirumani et al. reported that up to one-third of radiological manifestations of irAEs in patients treated with ipilimumab had no clinical manifestations [42]. A systematic description of radiological signs and of differential diagnoses will follow, with the aim of providing a guidance to the management of most significant irAEs. On the bases of incidence, severity and role of imaging we describe irAEs in the following anatomical districts: colon, liver, lung, endocrine system, and pancreas.

### 6.1. Colon

Besides dermatitis, colitis is the most frequent irAE [16]. Colitis is more common during treatment with anti–CTLA-4 antibodies [1]. Patients that develop toxicity may be asymptomatic; however, they are most frequently symptomatic with symptoms including diarrhea, abdominal pain, vomit, fever, occasionally associated with haematochezia, and/or endoscopic evidence of colon inflammation [4]. The current literature describes two main CT patterns, diffuse colitis, and segmental colitis, characterized by specific imaging features (Table 2).

**Figure 2:**
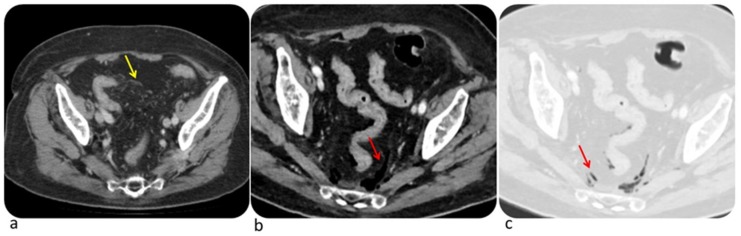
Complicated immune-related colitis in a 54-year-old man with metastatic melanoma treated first with ipilimumab then with Pembrolizumab. CT image shows mesenteric vessel engorgement (**a**, yellow arrow); CT performed two months later demonstrates free intraperitoneal air (**b**,**c**, red arrow) due to intestinal perforation. © Department of Radiology, Candiolo Cancer Institute—IRCCS. Turin.

**Figure 3:**
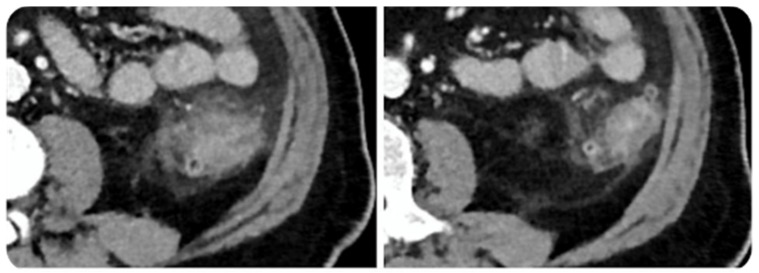
Complicated immune-related segmental colitis with diverticulosis pattern in a 76-year-old man with angiosarcoma and abdominal pain during Nivolumab treatment. CT images of two different slices show a segmental and circumferential descending colon wall thickening and pericolic fat stranding. © Department of Radiology, Candiolo Cancer Institute—FPO-IRCCs. Turin.

Examples of complicated immune-related colitis and segmental colitis associated with diverticulosis (SCAD) are shown in Figure 2 and Figure 3. According to Kim et al. early signs of ipilimumab-related colitis include the presence of mesenteric vessel engorgement at CT [5]. Conversely, they found that pneumatosis, bowel wall edema, halo, or target sign are more likely correlated with other etiologies of colitis [5]. Differential diagnosis must be considered with Crohn’s disease, ulcerative colitis, infectious colitis and pseudomembranous colitis. Crohn’s disease affects mainly terminal ileum with a patchy transmural distribution while ulcerative colitis increases intensity distally. Infectious colitis is most commonly localized in the right colon and is characterized by wall thickening with homogeneous enhancement while pseudomembranous colitis normally appears with marked circumferential or eccentric wall thickening [43]. Occasionally, the diagnosis of irAEs-related colitis is supported with biopsy which may show neutrophilic infiltrate, lymphocytic infiltrate, or a mix of both [44]. CT should be considered if toxic megacolon or intestinal perforation are suspected. 

### 6.2. Liver 

**Figure 4:**
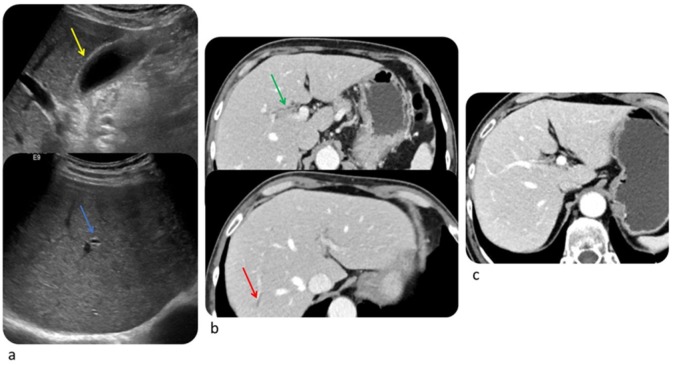
Immune related cholangitis in a 69-year-old female with metastatic melanoma treated with ipilimumab + pembrolizumab. Ultrasound shows layered gallbladder wall thickening likely representing gallbladder wall edema (**a**, yellow arrow) and hyperechogenicity of periportal space (**a**, blue arrows). Computed Tomography image shows mild wall thickness and contrast enhancement of the right hepatic duct (**b**, green arrow) and mild dilated intrahepatic bile duct (**b**, red arrow). Computed Tomography performed after steroid therapy demonstrates resolution (**c**). © Department of Radiology, Candiolo Cancer Institute—IRCCS. Turin.

The incidence of immune-related hepatotoxicity has been estimated to be 3–9% for ipilimumab, 0.7–1.8% for anti-PD-1/PD-L1, and greater for combined therapy [45,46]. Immune-related hepatitis generally occurs 6–14 weeks after the start of therapy [4] and presents with an asymptomatic elevation of ALT, AST, and total bilirubin (5–10% for monotherapy and 25–30% for combined therapy as ipilimumab + nivolumab) and less frequently with fever, fatigue, jaundice, and changes of stool color [1,24]. Kim at al described two histological pattern, acute hepatitis pattern where hepatocyte injury predominates, and biliary pattern where injury to bile ducts predominates [47]. CT and MRI may reveal hepatomegaly, periportal edema, periportal lymphadenopathy, periportal T2-hyperintensity, and attenuated liver parenchyma. A decreased attenuation of the liver parenchyma may obscure hepatic metastases and the appearance of new geographic areas of low attenuation may mimic metastases. After steroid therapy hepatomegaly and periportal lymphadenopathy generally resolve. US may show prominent periportal echogenicity and gallbladder wall edema (Figure 4) [47]. Differential diagnosis should be made with viral hepatitis, alcoholic liver disease, and idiopathic autoimmune hepatitis [43]. Therefore, in case of a diagnostic doubt viral serology, history of alcohol abuse, steatohepatitis, cirrhosis, and the presence of autoantibodies should be evaluated. Depending on the grade of toxicity it could be necessary to stop ICIs and to start steroid therapy, which usually brings to resolution of immune-related hepatitis in 4–6 weeks [3].

### 6.3. Lung

**Figure 5:**
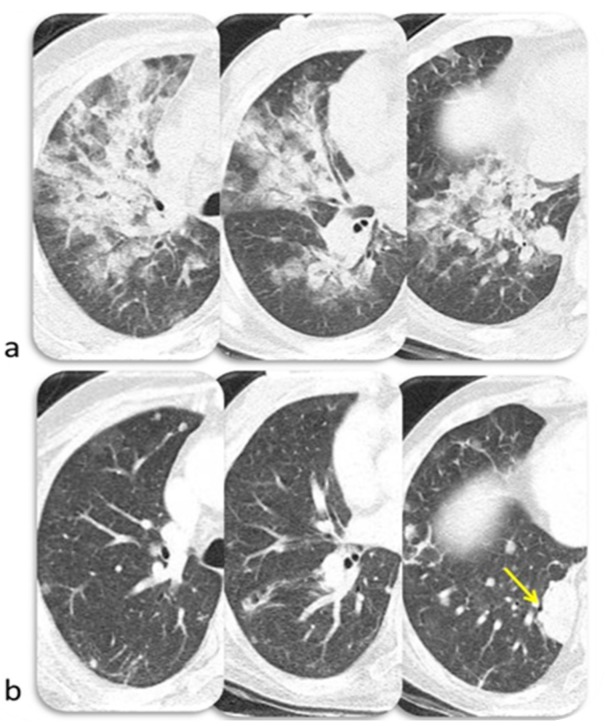
Immune related pneumonitis (pattern AIP/ARDS) in a 47-year-old woman with metastatic thymic carcinoma during treatment with pembrolizumab (**a**). CT image after 2 months of steroid therapy shows resolution of ARDS (**b**) and progressive disease (yellow arrow). © Department of Radiology, University of Pisa.

Lung toxicity has a variable onset and severity; uncommonly it may lead to a fatal event [1]. According to Wang DY et al., anti–PD-1/PD-L1–related fatalities occur most commonly from pneumonitis (35%) [48]. The median time to onset of immune-related pneumonitis is about 2–3 months after initiation of ICIs and its onset is anticipated in patients undergoing combined therapy or with non-small cell lung cancer [49]. However, it might occur at any point during treatment [50]. Pneumonitis has been identified in less than 1% of patients treated with anti–CTLA-4 antibody [49,51], in 1–6% of those treated with anti–PD-1/anti–PD-L1 (1–2% for melanoma, 3–6% for lung cancer) [52,53,54,55,56] and in 10% of patients during combined ICIs treatment [57,58]. Nishino et al. identified four CT patterns of pneumonitis in patients treated with PD-1 Inhibitor: Cryptogenic organizing pneumonia (COP), Non-specific interstitial pneumonia (NSIP), Hypersensitivity pneumonitis (HP), and Acute interstitial pneumonia/acute respiratory distress syndrome (AIP/ARDS) [59]. COP was the most common pattern; at CT it is characterized by patchy ground-glass opacities and consolidation in the subpleural region, or peribronchial. It is occasionally accompanied by the reversed “halo sign”. NSIP was the second most frequent pattern; at CT it presents with bilateral ground-glass opacities, with reticular opacities, traction bronchiectasis or bronchiolectasis, and minimal or absent honeycombing. It has a preferential basal distribution with subpleural sparing. HP at CT consists of centrilobular nodules and mosaic attenuation due to air trapping with an upper lobe–predominant distribution. The typical findings of AIP at CT are patchy bilateral ground-glass opacities with consolidation in the dependent lung, superimposed reticular densities (“crazy paving”) with a ventrodorsal and craniocaudal gradient. The end-stage lung disease with fibrosis is characterized by honeycombing and bronchiectasis [60,61]. An example of pembrolizumab-induced pneumonitis with an AIP pattern is shown in Figure 5. Clinical history may help to rule out pneumonitis with different etiology as other drug-induced pneumonitis, radiation pneumonitis, and bacterial pneumonia [43]. Radiation pneumonitis usually involves an area of the lung that is exposed to at least 30–40 Gray; in addition, such pneumonitis is not limited by interlobar fissures or by broncho-vascular structures and is characterized by ground glass opacities which may increase in density over time [62]. Bacterial pneumonia usually shows consolidations with air-bronchogram and is associated to pleural effusion; resistance to antibiotic therapy, negative sputum and bronchioalveolar lavage should support the exclusion of infectious etiology [63]. 

**Figure 6:**
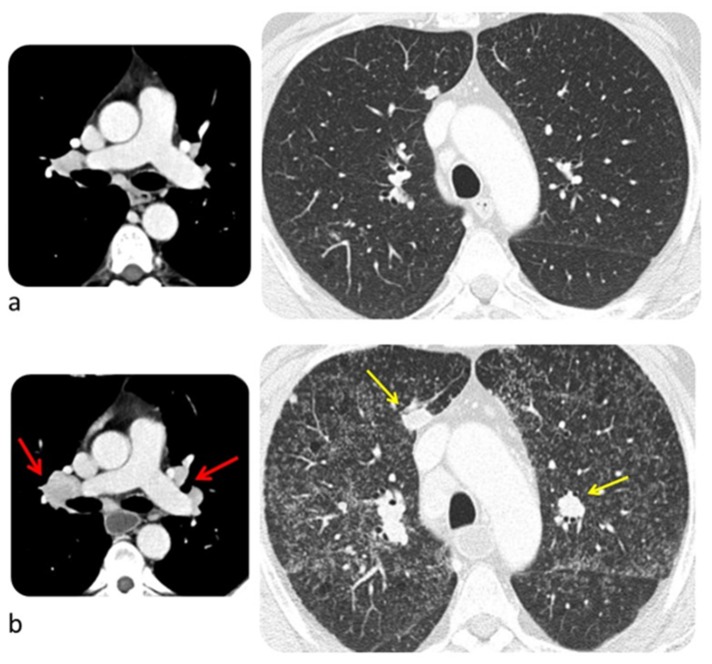
Sarcoid-like post immunotherapy granulomatosis in a 48-year-old man with metastatic melanoma. CT scan was performed before starting pembrolizumab (**a**) and two months later (**b**). The latter shows appearance of disseminated small nodules similar in size, enlarged hilar and mediastinal lymph nodes (red arrow), and progressive disease (yellow arrow). © Department of Radiology, Candiolo Cancer Institute—IRCCS. Turin.

Sarcoid-like post-immunotherapy granulomatosis is an irAE that most frequently involves lung but also other organs as hilar and mediastinal lymph nodes, and skin. It occurs in 5–7% of patients treated with ICIs, especially if they are affected by melanoma, with 6 months as a median time of onset [64]. Typical findings at CT are small nodules similar in size in a peribronchovascular distribution mostly in the upper lobes and symmetric hilar and mediastinal lymphadenopathy (Figure 6) [65]. Patients with post-immunotherapy granulomatosis are usually asymptomatic or may present with nonspecific skin lesions of erythema nodosum–like panniculitis. Alternatively, the patient may present with a triad of symptoms including uveitis, parotitis, and fever. It is associated with a favorable anti-tumor therapy response [64]. Post-immunotherapy granulomatosis must be distinguished from metastatic lymphadenopathy and lung metastases; the first generally is asymmetric, may have inhomogeneous enhancement and necrosis, while the latter usually is represented by nodules with different size [43]. Histologic examination of sarcoid-like granulomatosis of the lung includes non-necrotizing granulomatous inflammation [64,66].

### 6.4. Endocrine System 

Immune-related endocrine adverse events occur in up to one-third of patients treated with immune checkpoint inhibitors [67]. According to a meta-analysis conducted by Abdel-Rahman et al., patients with solid tumors that receive immune checkpoint inhibitors have a higher risk of hypophysitis (Relative Risk: 22.3), hypothyroidism (RR: 8.26), hyperthyroidism (RR: 5.48), and adrenal insufficiency (RR: 3.87). No differences in incidence and severity were detected among different categories of agents (CTLA-4 vs PD-1) or different cancer types [68]. Nevertheless, other studies demonstrated a major prevalence of thyroid disorders in patient treated with anti-PD-1 and a higher rate of pituitary disorders in those treated with anti CTLA-4 [69].

#### 6.4.1. Pituitary Gland

**Figure 7:**
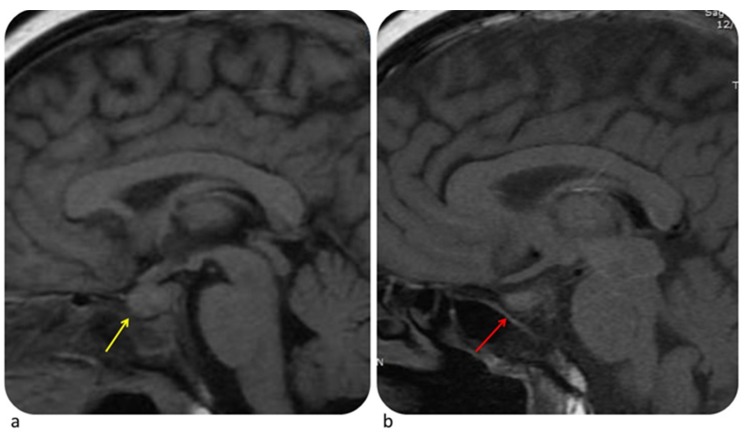
Hypophysitis in a 54-year-old woman with metastatic melanoma treated with ipilimumab. MRI performed at time of headache shows homogeneously enlarged pituitary gland (**a**, yellow arrow). Ipilimumab was stopped and patient was given hormone replacement therapy. MRI obtained one month later shows decrease in size of pituitary gland (**b**, red arrow). © Department of Neuroradiology, University of Pisa.

The Median time to onset of hypophysitis is 9 weeks. Two to 4% of patients treated with ICIs, especially with ipilimumab, develop hypophysitis [43]. Clinical signs of hypophysitis include headache, fatigue, hypothyroidism, hypogonadism, and hypocortisolism. Usually MRI shows moderate enlargement of the pituitary gland, with a convex aspect, enlargement of the stalk or infundibulum and homogeneous contrast enhancement (Figure 7). When assessing hypophysitis other endocrine disorders (pituitary adenoma, metastasis, lymphocytic hypophysitis) must be ruled out through imaging and clinical history [43]. Contrary to hypophysitis, pituitary adenoma appears as an asymmetric enlargement with heterogeneous contrast enhancement and loss of pituitary bright spot [70]. Pituitary metastasis occurs more frequently in melanoma, breast cancer, and lung cancer. Lymphocytic hypophysitis is an autoimmune pituitary disease so it may have similar features. However, the latter usually involves young women during pregnancy or postpartum period with headache, visual impairment, and ACTH deficiency [71]. Depending on the grade of toxicity it could be necessary to stop ICIs, start hormone replacement therapy (HRT) and, in some cases, start steroid therapy [3].

#### 6.4.2. Thyroid

The clinical or radiological suspicious of thyroid dysfunction needs confirmation with US. At US ICIs associated thyroiditis is similar to Hashimoto’s thyroiditis (diffusely enlarged gland, micronodular pattern, US-doppler evaluation showing normal, increased or decreased vascularity) [67]. In cases of thyroiditis, CT may show diffuse enlargement, appear hypodense and hypoenhancing. After switching from hyperthyroidism to hypothyroidism, the gland remains hypodense but decreases in size. The differential diagnosis includes thyroid metastasis, which are generally unifocal and tend to occur in patients with widespread metastatic disease from renal cell carcinoma, lung adenocarcinoma, breast cancer or melanoma [67,72].

### 6.5. Pancreas

**Figure 8:**
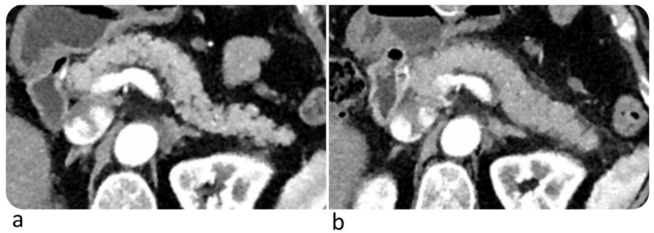
Complicated immune-related pancreatitis in a 61-year-old woman with metastatic melanoma treated with Nivolumab. CT image on arterial phase after 6° Nivolumab showed a normal pancreas (**a**); after 9° Nivolumab (4 months) CT showed a diffuse pancreatic enlargement associated with peripancreatic fat stranding (**b**). The patient was asymptomatic, but the laboratory test demonstrated an increase of lipase and amylase enzymes. © Department of Radiology, Candiolo Cancer Institute—FPO-IRCCs. Turin.

Immune-related pancreatitis are rare (incidence <1%) [73] and have been reported after an average of 3.8 months from initiation of therapy [42]. Patients with immune-related pancreatitis may be clinically asymptomatic or may present with upper abdominal pain. Increase in serum amylase and lipase may be observed. However, routine monitoring of such enzymes in asymptomatic individuals is not recommended [8,43]. Pancreatic enlargement with decreased enhancement and/or peripancreatic fat stranding may be observed at CT (Figure 8). Differential diagnosis with immunoglobulin G4-related autoimmune pancreatitis may be challenging. The latter is usually characterized by focal forms, may show a typical loss of the normal fatty lobulations, i.e., “sausage pancreas”, and may have simultaneous findings in multiple other organs (biliary, salivary, aortic, and retroperitoneal involvement) [43]; furthermore, immunoglobulin G4-related autoimmune pancreatitis often comes with upper abdominal pain and obstructive jaundice. Pancreatic atrophy due to immune checkpoint inhibitors has been reported [67]. 

## 7. PET-CT: Confounder or Problem-Solving?

Fluorodeoxyglucose positron emission tomography (FDG-PET) is the most sensitive method to detect inflammation from any cause, including from irAEs. Therefore, PET imaging could in principle provide beneficial information to clinicians in this setting. Furthermore, being it a whole-body modality, FDG-PET could allow localization of irAEs in multiple districts [74]. 

Mekki et al. found that 83% of patients with irAEs that underwent 18F-FDG PET-CT showed a high glucose uptake in relation with sarcoidosis-like syndrome, thyroiditis, hypophysitis, enterocolitis, and pancreatitis [40]. PET-detectable hepatitis, pneumonitis, arthritis, enthesitis, and myositis have also been reported [74]. 

Nobashi et al. noted that 64% of PET-detectable irAEs were asymptomatic [75]. Metabolic signs of irAEs may require careful monitoring and could suggest a change of therapy. Furthermore, since approximately 82% of patients had a complete response to immunotherapy on the final restaging scan, PET-detectable irAEs could be a favorable prognostic marker. Among all irAEs, thyroiditis was the earliest to onset. Therefore, an early increased-FDG uptake in thyroid may be a favorable indicator of response to immunotherapy [75]. 

Lang et al. reported that among 100 patients treated with ipilimumab for metastatic melanoma, 49 had increased-FDG uptake in the colon, 57% of whom with no clinical symptoms [76]. Conversely, Bronstein et al. suggested that clinically evident enterocolitis may not show morphological abnormalities on imaging. Indeed, only 5% of patients in the study (6 of 119) had evidence of colitis at CT with thickening of the colonic wall [77]. Hence, PET-CT is more sensitive than CT scans for the early detection of ICI-related colitis. However, considering all irAEs, Tirumani et al. demonstrated that among the total of 748 CT and 326 PET-CT scans reviewed, most of irAEs were detected on CT alone (83% on CT, 12% on PET/CT, 2% both on CT and on PET-CT) [42]. 

FDG-PET has the potential to detect irAEs. Hence, an integrated approach of irAEs including clinical, morphological and metabolic information could improve therapeutic decision-making in selected patients. Future studies on larger groups of patients and on a wider spectrum of malignancies are needed to validate PET imaging as a problem-solving examination in this context.

## 8. Less Frequent irAEs

Renal, cardiac, and neurological immune-related toxicity must be considered in patients under immunotherapy, although less frequent [1]. 

### 8.1. Renal Toxicity

The incidence of nephrotoxicity has been reported from less than 1% (monotherapy) to 4.9% (double immunotherapy) with a median time to onset of 3 months [3,26]. Although cross-sectional imaging is not the investigation of choice for immune-related nephropathy, CT imaging with focal or diffuse decreased enhancement of the renal parenchyma and renal pelvic thickening or cortical swelling has been reported as well as diffuse FDG uptake of the kidneys [12]. Renal toxicity is often detected with asymptomatic increase of serum creatinine [1]. The latter determinates the grade of severity and if grade 3–4 permanent withdrawal of ICIs and corticosteroids are recommended [13,26].

### 8.2. Cardiac Toxicity

Although rare (incidence <1%), cardiotoxicities are potentially fatal and can occur from 2 days to 15 months after start of ICIs [78]. The mechanisms of cardiotoxicity seem correlated to an autoimmune T-cell-mediated injury and to a non-inflammatory cardiomyocyte dysfunction in diseased hearts [78]. Risk factors for cardiac irAEs are the co-administration of an ICI with a cardiotoxic drugs or with another ICI and a previous cardiovascular or autoimmune disease. The most reported toxicities are conduction disease with heart block, ventricular arrhythmias, and myocarditis. The latter shows myocardial inflammation on MRI, PET/CT, or endomyocardial biopsy [78]. Acute myocardial infarction, non-inflammatory left ventricular systolic dysfunction, Takotsubo syndrome, and pericarditis can also occur [1,3,13,78]. Symptoms of cardiac disfunction, cardiac biomarker, and electrocardiogram determine the grade of toxicity. After grade 1, permanent withdrawal of ICIs, corticosteroids and infliximab in refractory cases are recommended [13,26].

### 8.3. Neurological Toxicity

The incidence of ICIs-related neurotoxicity is reported 3.8% for anti-CTLA4, 6.1% for anti-PD-1 and 12% for combined immunotherapy, with a median time to onset of 6 weeks [3,26]. High-grade neurological irAEs are rare (≤1%) [26]. Presentations range from nonspecific symptoms (fatigue, headache) to Guillain Barre’ syndrome, central and/or peripheral neuropathy, transverse myelitis, aseptic meningitis, myasthenia gravis, and encephalitis [1,3,79]. The latter two are the most related to fatality. Headache, encephalopathy and meningitis are the most reported events and have mainly low grade-severity [26]. The differential diagnosis includes progression of cancer, seizure activity, infection, and metabolic derangement. MRI, lumbar puncture, and nerve conduction studies help for rapid diagnosis. Although most neurological irAEs respond to high-dose steroids, some types need additional therapy (e.g., intravenous immunoglobulin for Gullain–Barré syndrome). Permanent discontinuation of ICIs is recommended for toxicity of grade 3–4 [3,13,26]. 

## 9. Future Directions and Conclusions

In the future imaging biomarkers could be developed to predict patients at higher risk of developing irAEs. To this point, initial research in the field of radiomics-based artificial intelligence are encouraging. In particular, Colen et al. were able to identify radiomics features at baseline CT discriminating patients who subsequently developed ICIs-related pneumonitis (100% accuracy; *p* = 0.0033) [80]. Despite the small number of patients included, the study shows the potential of radiomics to identify reliable predictors of irAEs and to stratify patients according to risk before therapy is initiated. Further studies on larger groups of patients and on a wider spectrum of irAEs are warranted to validate the clinical utility and generalizability of these predictive imaging biomarkers. Furthermore, imaging analysis could be combined with clinical and molecular data to yield more comprehensive clinical decision support systems.

In conclusion, health professionals, including radiologists, should be aware of the spectrum of adverse reactions related to the new cancer therapies, including therapy with ICIs, and should be able to recognize their clinical and radiological signs. Such signs if identified early after their onset, will immediately prompt therapy that improves patient outcome. Radiologists should also be aware that immunotherapy indications are evolving rapidly and that every year new drugs are introduced in clinical practice, which may be associated with new adverse events, prompting their continuous education. 

## Figures and Tables

**Table 1:** Approved Immune-Checkpoint Inhibitors Updated on March 2019 according to United States Food and Drug Administration (U.S. FDA) [6].

ICIMechanism of Action	Indication	
**Ipilimumab** **Anti-CTLA-4 antibody**	• Unresectable or Metastatic Melanoma • Adjuvant Treatment of Melanoma • Advanced Renal Cell Carcinoma • Microsatellite Instability-High (MSI-H) or Mismatch Repair Deficient (dMMR) Metastatic Colorectal Cancer	
**Pembrolizumab** Anti-PD-1 antibody	• Melanoma • Non-Small Cell Lung Cancer • Small Cell Lung Cancer • Head and Neck Squamous Cell Cancer • Classical Hodgkin Lymphoma • Primary Mediastinal Large B-Cell Lymphoma • Urothelial Carcinoma • Microsatellite Instability-High Cancer	• Gastric Cancer • Esophageal Cancer • Cervical Cancer • Hepatocellular Carcinoma • Merkel Cell Carcinoma • Renal Cell Carcinoma • Endometrial Carcinoma
**Nivolumab** Anti-PD-1 antibody	• Unresectable or Metastatic Melanoma • Adjuvant Treatment of Melanoma • Metastatic Non-Small Cell Lung Cancer • Small Cell Lung Cancer • Advanced Renal Cell Carcinoma • Classical Hodgkin Lymphoma	• Squamous Cell Carcinoma of the Head and Neck • Urothelial Carcinoma • Microsatellite Instability-High or Mismatch Repair Deficient Metastatic Colorectal Cancer • Hepatocellular Carcinoma.
**Ipilimumab + nivolumab**	• Unresectable or metastatic melanoma	
**Atezolizumab** Anti-PD-L1 antibody	• Urothelial Carcinoma • Non-Small Cell Lung Cancer • Locally Advanced or Metastatic Triple-Negative Breast Cancer • Small Cell Lung Cancer	
**Avelumab** Anti-PD-L1 antibody	• Metastatic Merkel Cell Carcinoma • Locally Advanced or Metastatic Urothelial Carcinoma • Advanced Renal Cell Carcinoma	
**Durvalumab** Anti-PD-L1 antibody	• Urothelial Carcinoma • Non-Small Cell Lung Cancer	
**Cemiplimab** Anti-PD-1 antibody	• Metastatic Cutaneous Squamous Cell Carcinoma (CSCC) • Locally Advanced CSCC	

**Table 2:** Two computed tomography (CT) patterns of immune-mediated colitis.

Diffuse Colitis	Segmental Colitis
Diffuse colonic wall thickening Mucosal hyperenhancement Mesenteric vessel engorgement With or without fluid filled distended colon	Segmental colonic wall thickening Pericolic fat stranding Mucosal hyperenhancement Mesenteric vessel engorgement Often superimposed to diverticulitis, named also “segmental colitis associated with diverticulosis”, characterized by mild systemic symptoms and absence of fecal bacterial/leukocyte

## Abbreviations

AIP/ARDS	Acute Interstitial Pneumonia/Acute Respiratory Distress Syndrome
ALT	Alanine Transaminase
ASCO	American Society of Clinical Oncology
AST	Aspartate aminotransferase
COP	Cryptogenic Organizing Pneumonia
CT	Computed Tomography
CTCAE	Common Terminology Criteria for Adverse Events
CTLA-4	Cytotoxic T-Lymphocyte–Associated Antigen 4
EMA	European Medicines Agency
ESMO	European Society for Medical Oncology
FDG-PET	Fluorodeoxyglucose-Positron Emission Tomography
FT4	Free Thyroxine
HP	Hypersensitivity Pneumonitis
HRT	Hormone Replacement Therapy
ICIs	Immunotherapy with Checkpoint Inhibitors
irAEs	immune-related Adverse Events
MRI	Magnetic Resonance Imaging
NCCN	National Comprehensive Cancer Network
NCI-CTCAE	National Cancer Institute - Common Terminology Criteria for Adverse Events
NSIP	Non-Specific Interstitial Pneumonia
PD-1	Programmed Death 1
PD-L1	Programmed Death-Ligand 1
RR	Relative Risk
SCAD	Segmental Colitis Associated with Diverticulosis
TSH	Thyroid-Stimulating Hormone
U.S. FDA	United States Food and Drug Administration
US	Ultrasound
